# Nighttime lighting effects on perceived restoration depend on landscape type: evidence from classical garden landscapes and modern park landscapes

**DOI:** 10.3389/fpsyg.2026.1958901

**Published:** 2026-10-23

**Authors:** Dongyu Liu, Xue Gong, Yuting Dai

**Affiliations:** 1 City Graduate School, City University Malaysia, Kuala Lumpur, Malaysia; 2 College of Horticulture, China Agricultural University, Beijing, China; 3 School of Design and Innovation, Jiangsu University of Architectural Technology, Xuzhou, China; 4 Artinstitute of Xinjiang Normal University, Urumqi, China

**Keywords:** artificial light, environmental psychology, natural light, nighttime landscapes, restorative environments

## Abstract

**Background:**

Urban green spaces can support perceived restoration, but nighttime responses may depend on how lighting interacts with landscape context. Previous research has focused mainly on daytime environments or treated nighttime lighting as an independent factor, leaving this interaction insufficiently understood.

**Methods:**

A 2 × 2 between-participants experiment compared sampled classical-garden scenes from Suzhou and modern-park scenes from Xuzhou under natural nighttime light and scene-specific simulated artificial lighting. 200 participants were randomly assigned to four conditions (50 per condition), with each participant evaluating five images from their assigned condition. Self-reported responses were assessed using an adapted Short-version Revised Restoration Scale, and six observable landscape characteristics were rated by 15 experts. Trial-level linear mixed-effects models included participant and base-scene random intercepts. The primary analysis tested the landscape type × lighting interaction for the overall score.

**Results:**

Across lighting conditions, the sampled classical-garden scenes had higher descriptive overall scores than the sampled modern-park scenes (7.14 ± 0.52 vs. 5.75 ± 0.42), although landscape type was confounded with city. The primary interaction was −0.544 points (95% CI [−0.792, −0.295], *p* < 0.001). In the sampled classical-garden scenes, artificial lighting was associated with lower overall scores than natural nighttime light (difference = −0.471, 95% CI [−0.647, −0.295], adjusted *p* < 0.001), whereas the corresponding contrast in the sampled modern-park scenes was small and uncertain (0.073, 95% CI [−0.103, 0.248]).

**Conclusion:**

Nighttime lighting responses differed between the two sampled scene sets, suggesting that lighting should be evaluated in relation to landscape context. These findings concern self-reported responses to specific photographic scenes and do not establish general differences between classical gardens and modern parks or the effectiveness of particular lighting designs.

## 1 Introduction

Rapid urbanization, increasingly intensive work patterns, and the extension of daily activities into evening hours have increased concerns about stress, mental fatigue, and reduced opportunities for psychological recovery in cities (Baumann et al., 2020; Berto, 2005; Bowler et al., 2010). Urban green spaces are therefore increasingly understood not only as ecological infrastructure but also as settings that can support emotional regulation, stress reduction, and attentional renewal (Gascon et al., 2015; McMahan and Estes, 2015; Octaviani et al., 2025; Parvez, 2026). Attention Restoration Theory proposes that environments characterized by fascination, being away, extent, and compatibility allow depleted directed-attention resources to recover, whereas Stress Reduction Theory emphasizes rapid affective and physiological responses to non-threatening natural settings (Kaplan, 1995; Ulrich et al., 1991). Experimental and field studies have provided convergent evidence that exposure to natural environments can improve mood, attention, and stress-related outcomes (Hartig et al., 2003, 1997).

The restorative value of an urban green space, however, cannot be inferred from the presence of greenery alone. Restorative responses vary with the perceptual and spatial qualities of a setting, including vegetation structure, species richness, openness, refuge, visual access, maintenance, water, terrain, and the arrangement of built elements (Grahn and Stigsdotter, 2010; Nordh et al., 2009; Peschardt and Stigsdotter, 2013; Tabrizian et al., 2018). Perceived sensory dimensions such as nature, refuge, serenity, and prospect have been associated with stress recovery, while studies of small urban parks show that vegetation, hardscape composition, and spatial organization can predict perceived restorativeness. More recent work further indicates that vegetation structure, color diversity, and multisensory landscape quality influence preference and restoration in interdependent rather than purely additive ways (Hao et al., 2024; Korpilo et al., 2024; Liu et al., 2024). These findings suggest that landscape type may function as an integrated perceptual context within which individual features acquire restorative meaning.

Chinese classical gardens provide a particularly relevant context for examining this proposition. In contrast to many modern parks, which commonly prioritize openness, circulation, multifunctional use, and broad visual accessibility, classical gardens organize experience through enclosure, framed views, sequential transitions, layered vegetation, terrain variation, water, and the close integration of architecture with natural elements (Guo et al., 2025; Li et al., 2024; Yu and Ostwald, 2018; Zhang et al., 2024; Zheng et al., 2024). Their spatial composition produces gradual visual disclosure and shifting relationships between foreground and background, potentially supporting fascination, psychological distance, contemplation, and sustained engagement. Empirical studies in Suzhou have reported restorative experiences in classical gardens and have linked such outcomes to esthetic engagement, cultural atmosphere, privacy, spatial scale, and activity settings (Liu et al., 2022; Sun et al., 2023). Nevertheless, direct controlled comparisons between classical garden landscapes and modern park landscapes remain limited, particularly under nighttime conditions (Haans and de Kort, 2012; Zhao et al., 2023a).

Nighttime introduces an additional layer of complexity because reduced illumination changes what can be seen, how spatial boundaries are interpreted, and which landscape features become salient (Boyce et al., 2000; Bullough et al., 2020). Artificial light at night can extend the usable hours of green spaces and improve orientation and perceived safety, yet it can also produce glare, visual fragmentation, excessive alertness, and loss of darkness, privacy, or mystery. Consequently, the psychological effects of lighting cannot be represented adequately by illuminance alone. A recent field study of urban green spaces found that high visual accessibility at night was associated with stronger restoration and that lighting position and distribution were consequential: path and foreground lighting could weaken fascination and being away, whereas background lighting and more scattered or uniform distributions could strengthen extent, compatibility, and restorative experience (Gao and Zhu, 2025). This evidence places the spatial organization of light, rather than brightness in isolation, at the center of restorative nightscape design.

Related nighttime studies reinforce this context-sensitive interpretation. Background lighting can improve preference by increasing legibility and perceived safety, while excessive foreground or path lighting may reduce preference (Lis et al., 2023). Adding ambient light does not produce a uniform benefit across park settings; its effects on safety, fascination, contemplation, and willingness to remain can reverse according to whether a space is open, semi-open, or enclosed (Lis et al., 2024). Walkway-lighting research likewise shows that scene content and the focus of illumination jointly shape perceived restorativeness, fear, and preference (Nikunen et al., 2014). Other experimental evidence demonstrates that nighttime lighting conditions affect emotional responses and perceived safety (Himschoot et al., 2024; Masullo et al., 2022), and that perceived spaciousness and lighting uniformity depend on the relationship between light patterns and surrounding spatial form (Wänström Lindh and Billger, 2021; Wänström Lindh and Jägerbrand, 2021). At the same time, growing concern over artificial light at night has prompted calls to treat darkness as a component of green and blue infrastructure rather than as an absence to be eliminated (Sordello et al., 2022).

Taken together, the literature suggests that nighttime restoration may emerge from compatibility between landscape structure and lighting configuration (Nikunen and Korpela, 2012, 2009; Trop et al., 2023; Zhao et al., 2023b). Natural nighttime lighting may preserve continuity, depth, and subdued atmospheric relationships in some landscapes, whereas carefully positioned artificial lighting may improve visual access, orientation, or perceived safety in others (Markvica et al., 2019; Nasar and Bokharaei, 2017; Portnov et al., 2020). The same intervention may therefore enhance restoration in one spatial context but weaken it in another by revealing, concealing, or visually isolating different landscape components. This possibility is especially important for classical garden landscapes, whose experiential qualities depend on layered composition, framed views, and controlled visual sequences, but it has rarely been examined against modern park landscapes within a consistent experimental framework.

Three gaps follow from this evidence. First, restorative-environment research remains dominated by daytime exposure, while nighttime studies have focused more often on safety, preference, or lighting performance than on multidimensional perceived restoration (Kostić and DJokić, 2014). Second, the restorative potential of classical garden landscapes and modern park landscapes has not been sufficiently compared under controlled nighttime conditions. Third, limited evidence explains whether lighting effects vary by landscape type or how observable landscape characteristics contribute within different landscape–lighting contexts. Addressing these gaps requires an approach that treats lighting as a contextual moderator of landscape perception rather than as an independent technical variable.

Accordingly, this study used controlled visual stimuli in a 2 × 2 between-participants design to examine classical garden landscapes and modern park landscapes under natural nighttime lighting and artificial lighting. It aimed to (1) compare perceived restoration between the two landscape types; (2) determine whether the effect of lighting differed between landscape types; and (3) explore, at the image level, the context-specific associations between six expert-rated landscape characteristics and emotional, physiological, cognitive, behavioral, and overall restoration within each of the four landscape type × lighting conditions. By integrating landscape type, lighting condition, and image-level landscape characteristics, the study seeks to clarify how nocturnal restorative experience varies across landscape–lighting contexts and to provide evidence for landscape-specific nighttime green-space design.

## 2 Materials and methods

### 2.1 Experimental design

To systematically investigate perceived restoration in nighttime landscapes under different environmental conditions, this study adopted a controlled image-based experimental design. Photographic stimuli provided a consistent set of visual scenes for evaluation and enabled matched comparisons between lighting conditions while preserving scene composition. Photographic stimuli have been used in previous research on landscape perception and environmental preference (Stamps, 1990; Velarde et al., 2007).

As shown in Figure 1, the study used a 2 × 2 between-participants design crossing sampled landscape set with lighting condition. The landscape sets comprised five classical-garden scenes from Suzhou and five modern-park scenes from Xuzhou, each presented under natural nighttime lighting or artificial lighting. Their combination resulted in four experimental conditions: classical garden landscapes under natural nighttime lighting (CL-NL), classical garden landscapes under artificial lighting (CL-AL), modern park landscapes under natural nighttime lighting (MD-NL), and modern park landscapes under artificial lighting (MD-AL). Landscape set was a sampled grouping rather than an experimentally manipulated property and was completely confounded with city; comparisons were therefore restricted to the sampled scenes.

**Figure 1:**
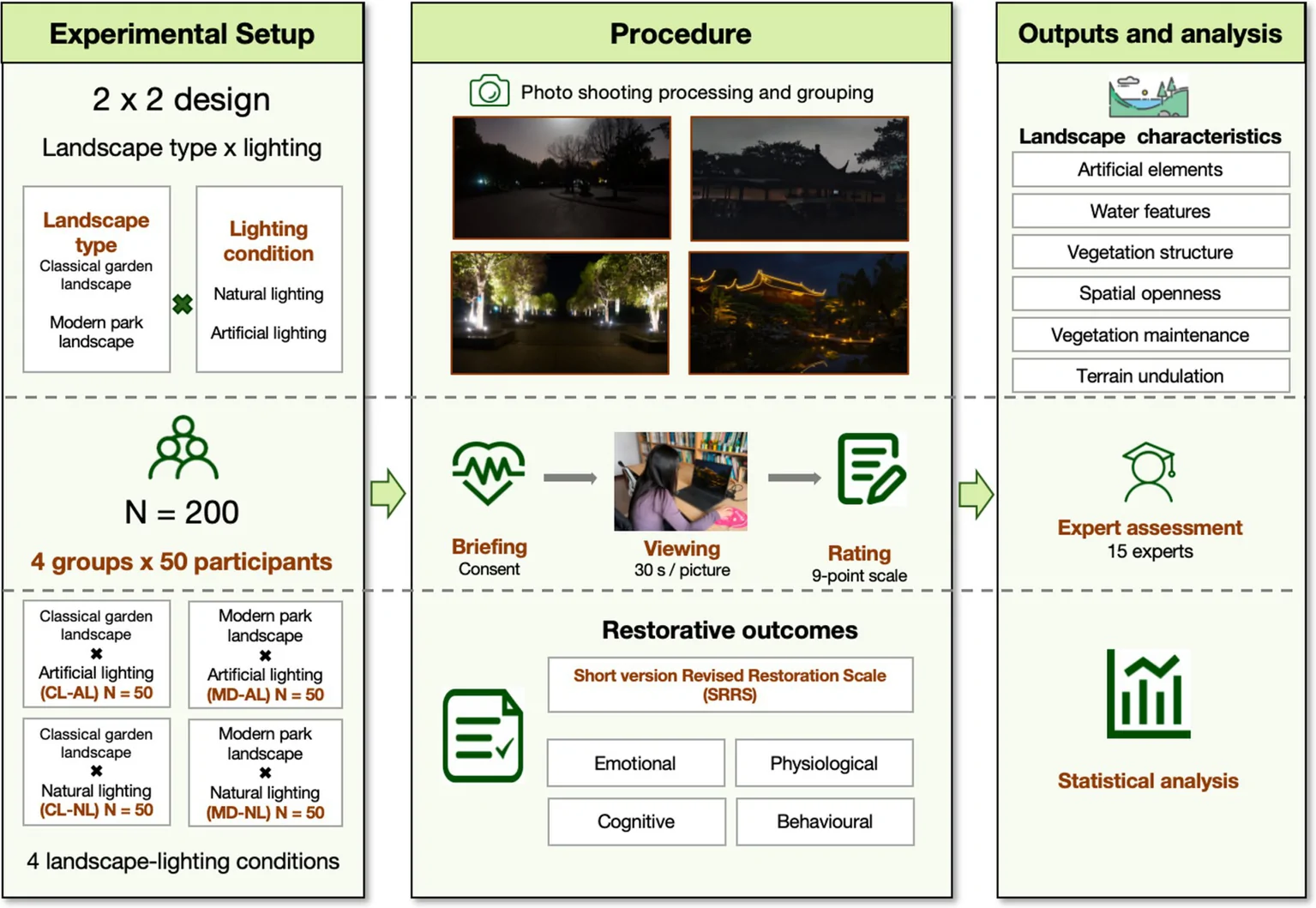
Research framework.

The stimulus set comprised 20 images derived from 10 base scenes, with each base scene represented by a matched natural-light and artificial-lighting pair. Participants were randomly assigned to one experimental condition and viewed the five photographs within that condition. Each participant provided five repeated image evaluations within one assigned condition, while each base scene was paired across lighting conditions.

Within each assigned condition, the five images were presented in a randomized order to reduce order effects. Participants evaluated each image separately according to their immediate impression and were not instructed to compare images directly.

During revision, overall perceived restoration was designated as the primary outcome, and the landscape set × lighting interaction as the primary hypothesis. Dimension-specific analyses were treated as secondary.

Fifteen experts separately rated six observable landscape characteristics in each of the 20 images using predefined ordinal criteria. Associations between these expert-rated characteristics and image-level mean overall perceived-restoration scores were retained solely as descriptive, hypothesis-generating observations in the Supplementary material.

### 2.2 Visual stimuli and landscape characteristics

#### 2.2.1 Visual stimuli collection

Suzhou and Xuzhou were selected within the same province, Jiangsu, China (Figure 2), to provide a shared provincial context for sampling classical-garden and modern-park scenes. Suzhou was chosen to represent Chinese classical garden landscapes (Song et al., 2025; Wang et al., 2014; Yu and Ostwald, 2018), whereas Xuzhou was selected to represent modern park landscapes. Five representative sites were selected in each city, resulting in 10 matched base scenes across the two landscape types.

**Figure 2:**
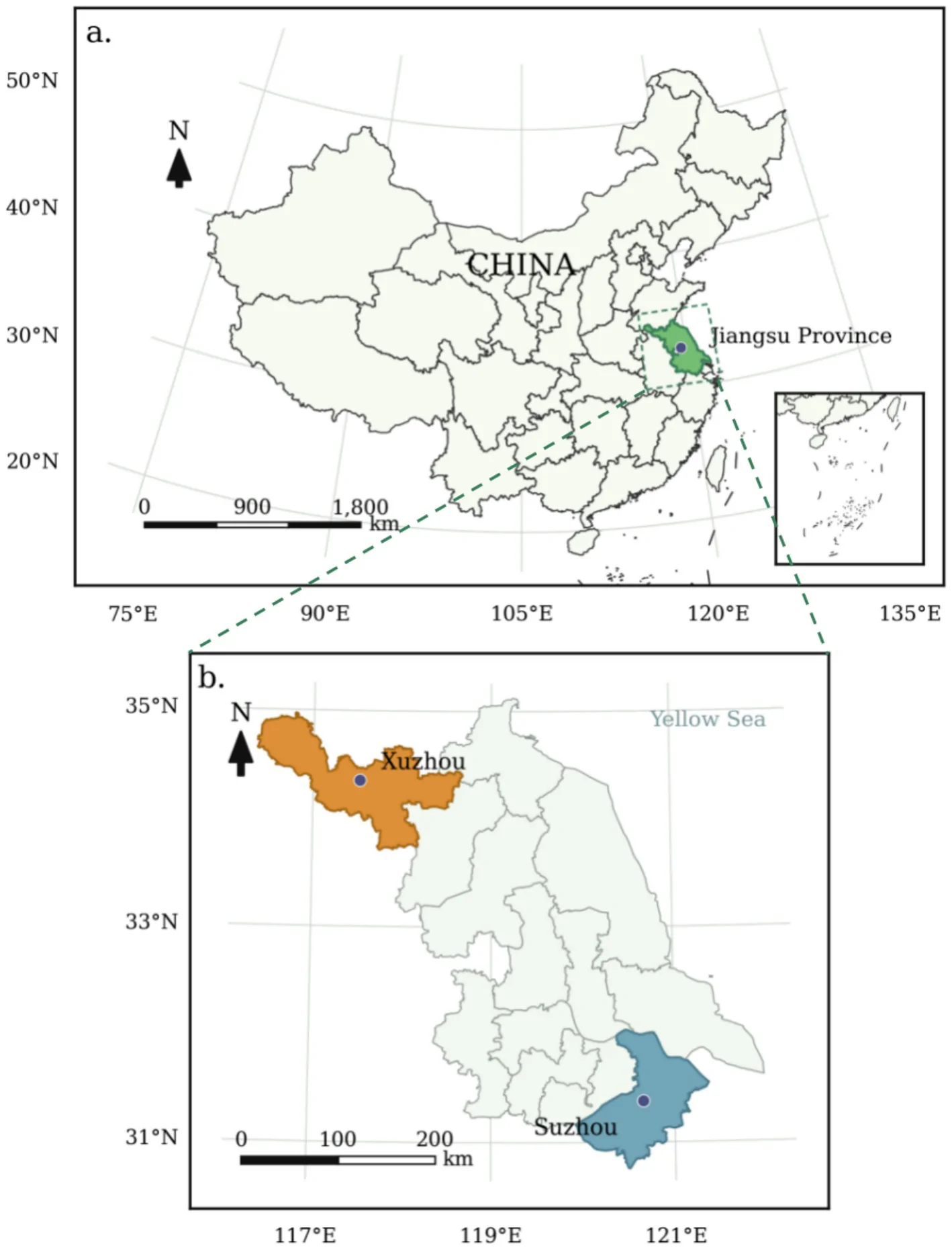
Geographical location of the study areas in Jiangsu Province, China.

All base photographs were captured during summer 2025 on clear, full-moon nights between 22:00 and 01:00, after the main park lighting had been switched off. Photographs were taken from fixed viewpoints approximately 1.5 m above ground, with the camera held level and identical camera settings, and were cropped to a common size before processing. These procedures reduced variation in photographic technique, weather, and lunar phase but did not ensure equivalent ambient illumination across sites. The natural-light condition could therefore include residual sky glow and surrounding urban light.

Author DL conducted field visits before and after the park lights were switched off and used Adobe Photoshop to create an artificial-lighting version of each natural-light photograph. The observed site lighting served as a visual reference for the placement of simulated light sources and the appearance of illuminated areas, with the aim of approximating the existing nighttime scene. Editing was restricted to lighting-related changes, preserving the viewpoint, spatial composition, and scene content within each pair. These images represented scene-specific visual simulations rather than photometrically calibrated reproductions or uniform changes in lighting intensity, color, or spatial distribution. The final stimulus set comprised 20 photographs arranged in 10 matched pairs, with five pairs per landscape type (Figure 3).

**Figure 3:**
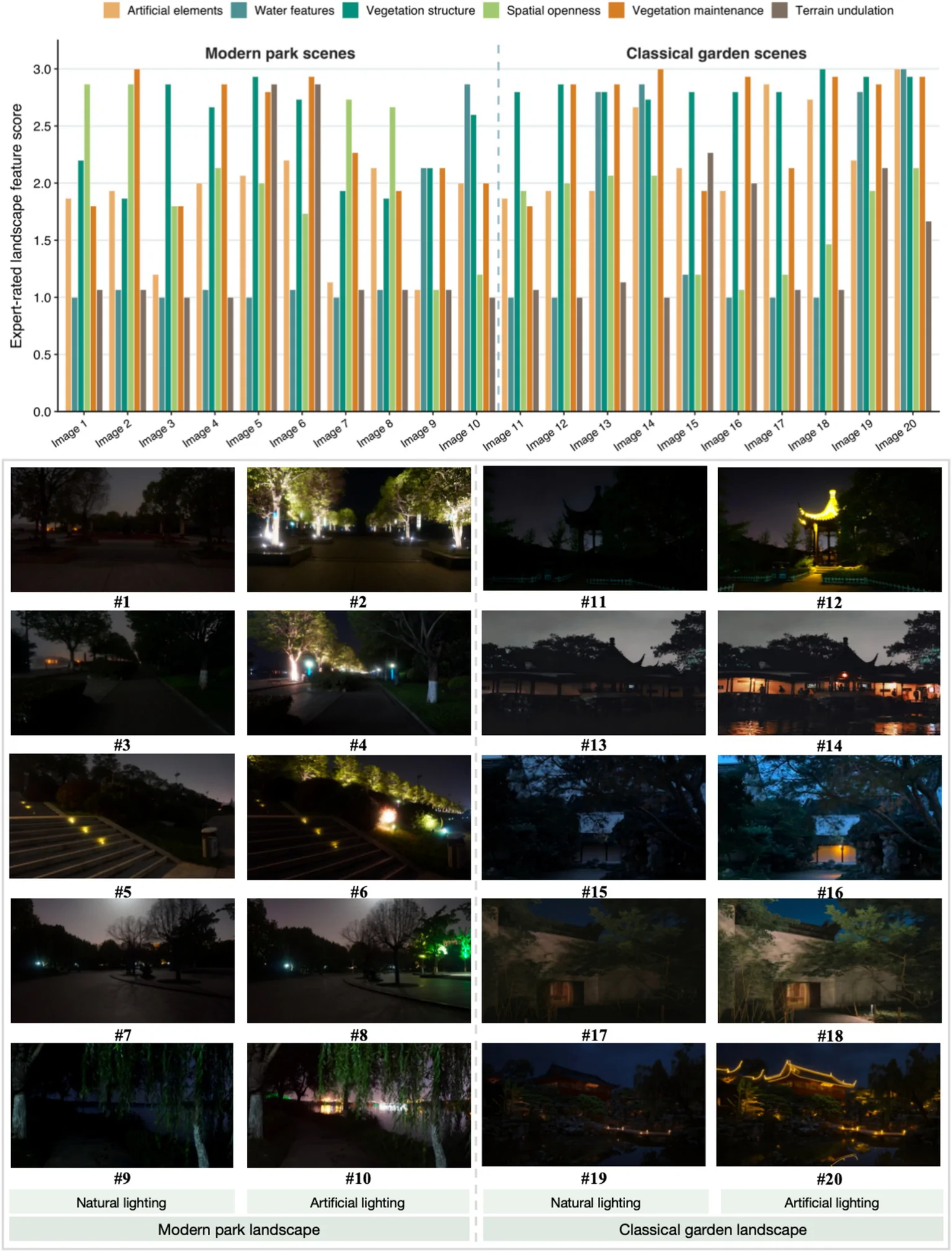
Landscape characteristics and experimental grouping of the photographic stimuli.

#### 2.2.2 Landscape characteristics quantification

To quantify the observable landscape characteristics of the photographic stimuli, six landscape characteristics were selected based on previous studies on landscape perception, visual preference, and restorative environments (Daniel and Meitner, 2001; Herzog et al., 2003; Huang et al., 2021; Korpela et al., 2008; Zhao and Huang, 2021). Following a review of the relevant literature, two authors (XG and DL) independently identified the landscape characteristics considered most relevant to perceived restoration in nighttime garden environments. Any disagreements were resolved through discussion with a third reviewer (YD). The final assessment included six characteristics: the proportion of artificial elements, water features, vegetation coverage structure, spatial openness, vegetation maintenance, and terrain undulation.

Fifteen experts independently evaluated all 20 photographs. The natural-light and artificial-lighting versions of each base scene were rated separately. Ratings reflected characteristics as visually discernible in each image and could therefore vary with lighting-dependent visibility, despite unchanged underlying scene geometry and composition (Koenderink et al., 1996; Wänström Lindh and Billger, 2021). The expert panel consisted of researchers and professionals holding at least a master’s degree in landscape architecture, urban and rural planning, architecture, or related disciplines. The photographs were presented in a randomized order to minimize potential order effects. The assessment focused on expert-rated, observable landscape characteristics and did not include perceived restoration. The complete assessment criteria and scoring form are provided in Supplementary Table S1.

Each characteristic was evaluated using a three-point ordinal scale according to predefined criteria. For example, the proportion of artificial elements was classified as occupying 0–20%, 20–50%, or more than 50% of the photograph, whereas the remaining characteristics were rated from low or simple conditions to high or complex conditions. For each photograph, ratings from the 15 experts were averaged to obtain a descriptive score for each landscape characteristic. These image-level mean scores were subsequently used to explore associations between expert-rated landscape characteristics and overall perceived restoration.

### 2.3 Questionnaire and psychological measures

Perceived restoration was measured using an adapted version of the eight-item Short-version Revised Restoration Scale (SRRS) developed by Han (2003). The original scale was designed to assess restorative responses to environmental scenes across four dimensions: emotional, physiological, cognitive, and behavioral restoration. The Chinese questionnaire retained the original eight-item grouping and the four-dimension labels: emotional, physiological, cognitive, and behavioral (Table 1). The original English items and the Chinese wording administered in this study are provided in Supplementary Table S2. In this study, these dimensions represent self-reported emotional responses, physiological sensations, cognitive engagement, and intentions to revisit or stay longer, respectively. The two physiological items were reverse-scored, so higher scores indicate lower endorsement of faster breathing and sweating. The overall score was interpreted as a composite of self-reported responses to the presented scene.

**Table 1:** Items and scoring of the short-version revised restoration scale.

Dimension	Item	Statement	Scoring*
Emotional restoration	Q1	I feel cheerful.	1–9
	Q2	I feel relaxed.	1–9
Physiological restoration	Q3	My breathing is becoming faster.	1–9
	Q4	My hands are sweating.	1–9
Cognitive restoration	Q5	I am interested in the presented scene.	1–9
	Q6	I am attentive to the presented scene.	1–9
Behavioral restoration	Q7	I would like to visit this place more often.	1–9
	Q8	I would like to stay here longer.	1–9

*Participants rated each item on a nine-point Likert scale ranging from 1 (totally disagree) to 9 (totally agree). Q3 and Q4 were reverse-coded as 10 minus the original response.

Before rating each image, participants were instructed to imagine themselves in the presented scene and indicate the extent to which they agreed with each statement. All items were rated on a nine-point Likert scale ranging from 1 (totally disagree) to 9 (totally agree). Each dimension contained two items. Because the two physiological items describe stress-related responses, their scores were reverse-coded as 10 minus the original response. The score for each dimension was calculated as the mean of its two corresponding items, and the overall perceived-restoration score was calculated as the mean of the four-dimension scores. Higher scores therefore indicated greater perceived restoration. The questionnaire also collected participants’ age and gender.

### 2.4 Participants and experimental procedure

An *a priori* power analysis was conducted using G*Power 3.1 for a fixed-effects 2 × 2 between-participants analysis of variance. Assuming a medium effect size (*f* = 0.25), *α* = 0.05, power = 0.80, one numerator degree of freedom, and four experimental groups, the required total sample was 128. The final sample included 200 valid participants and therefore exceeded this requirement.

In October 2025, 200 college students were recruited through online posters and offline publicity at a university in Jiangsu Province, China. All participants reported normal or corrected-to-normal vision and no known visual impairments. Each experimental condition included 50 participants. Participation was voluntary, written informed consent was obtained before the experiment, and all responses were collected anonymously. Participants were aged 19–30 years (mean ± SD: 24.48 ± 3.46); 104 (52.0%) were male and 96 (48.0%) were female. Participant characteristics overall and by condition are reported in Table 2. All 200 participants completed the questionnaire and were included in the analysis; there were no incomplete responses or participant exclusions. Each participant received a small gift upon completion.

**Table 2:** Participant characteristics overall and by randomized condition.

Characteristic	Overall	Classical–natural	Classical–artificial	Modern–natural	Modern–artificial
Participants, n	200	50	50	50	50
Age, mean ± SD, years	24.48 ± 3.46	24.42 ± 3.48	24.54 ± 3.46	24.42 ± 3.51	24.54 ± 3.49
Age range, years	19–30	19–30	19–30	19–30	19–30
Male, *n* (%)	104 (52.0)	26 (52.0)	26 (52.0)	26 (52.0)	26 (52.0)
Female, *n* (%)	96 (48.0)	24 (48.0)	24 (48.0)	24 (48.0)	24 (48.0)

An allocation sequence generated using random numbers was implemented to assign participants to four landscape–lighting conditions, with 50 participants per condition. Researchers administering the study were aware of participants’ assigned conditions and the study hypotheses; no researcher blinding was implemented (Figure 4).

**Figure 4:**
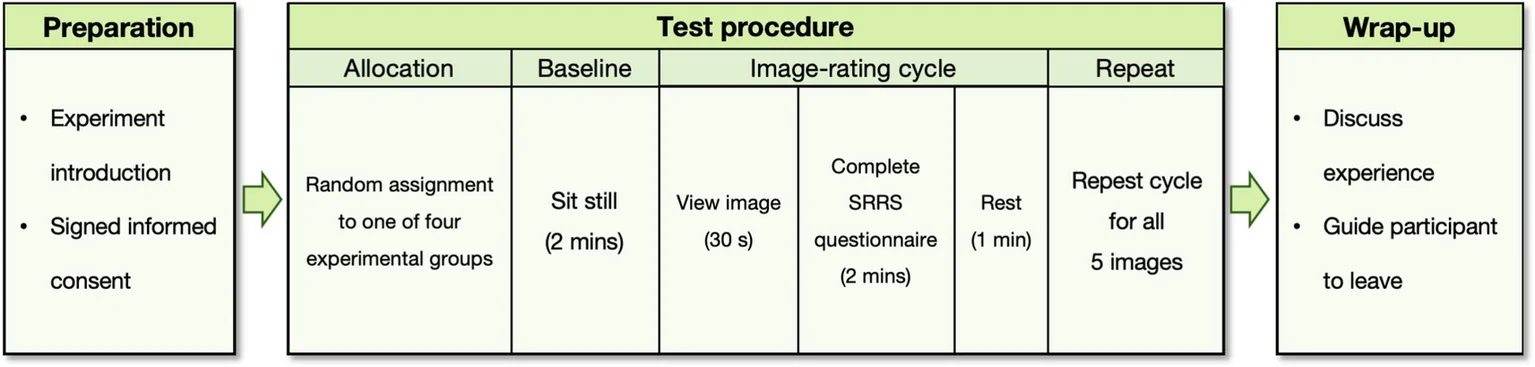
Experimental procedures.

The experiment was conducted individually in a controlled indoor environment with stable ambient lighting and minimal external disturbance. After receiving an introduction to the experiment and providing informed consent, each participant was randomly assigned to one of four experimental conditions. Participants then sat quietly for 2 min before beginning the image-rating task. Each participant viewed five nighttime landscape images corresponding to the assigned condition, presented in a randomized order.

Each image was displayed for 30 s and was immediately followed by completion of the eight-item SRRS questionnaire. Participants then rested for 1 min before viewing the next image. This viewing, evaluation, and rest cycle continued until all five images had been assessed. At the end of the session, participants briefly discussed their experience with the researcher and were guided from the experimental room.

### 2.5 Data analysis

All analyses were conducted using R version 4.5.2. All 1,000 image evaluations from 200 participants were retained; no item responses were missing, and no imputation was required. Psychometric analyses accounted for repeated evaluations within participants. Within-participant inter-item correlations and Spearman–Brown reliability were calculated for each two-item dimension, with 95% confidence intervals from 2,000 participant-level bootstrap samples stratified by condition. Two-level confirmatory factor analyses evaluated one-factor and correlated four-factor within-participant structures, allowing unrestricted between-participant covariances. Nine-point responses were treated as approximately continuous using robust maximum likelihood; both model fit and solution admissibility were assessed. Within-participant alpha and omega were estimated for the overall score, with image-mean-adjusted analyses as a sensitivity check. These analyses did not provide a fully crossed participant-and-scene psychometric evaluation.

During revision, the overall score and landscape type × lighting interaction were designated as the primary outcome and hypothesis; this choice was not preregistered. Linear mixed-effects models retained five evaluations per participant and included landscape type, lighting, and their interaction as fixed effects, with participant and base-scene random intercepts. Paired natural-light and artificial-lighting images shared a base-scene identifier. Models used restricted maximum likelihood and Kenward–Roger inference. The primary interaction compared the artificial-lighting minus natural-light contrasts between the two sampled scene sets. Its two-sided test used *α* = 0.05; Holm correction was applied jointly to 14 secondary tests comprising four dimension-specific interactions and 10 within-set lighting contrasts. Estimates, pointwise 95% confidence intervals, and *p* values were reported. Diagnostics addressed residual distributions, condition-specific variability, convergence, and singularity; flagged observations were retained. Sensitivity analyses allowed scene-specific lighting slopes, omitted individual base scenes, and adjusted for presentation order. Inference was restricted to the sampled scenes because landscape type and city were completely confounded.

Associations between six expert-rated landscape characteristics and overall perceived restoration were examined descriptively at the image level. Pearson correlations were calculated separately within the four landscape–lighting conditions, with five images per condition. Benjamini–Hochberg correction was applied across all 24 correlations. These analyses were treated solely as hypothesis-generating, without inferential claims about landscape predictors. Expert agreement was summarized using mean pairwise linearly weighted Cohen’s kappa, with 95% confidence intervals obtained by resampling paired base scenes. Uncertainty around image-level mean feature ratings was estimated using 95% bootstrap confidence intervals obtained by resampling experts.

## 3 Results

### 3.1 Differences in perceived restoration between classical garden landscapes and modern park landscapes

Across lighting conditions, participant-average scores were consistently higher for the sampled classical-garden scenes than for the sampled modern-park scenes across all four dimensions (Figures 5 and Supplementary Table S4): emotional restoration (mean ± SD: 7.23 ± 0.56 vs. 5.82 ± 0.47), physiological restoration (7.13 ± 0.56 vs. 5.71 ± 0.48), cognitive restoration (7.24 ± 0.55 vs. 5.87 ± 0.49), and behavioral restoration (6.95 ± 0.57 vs. 5.61 ± 0.44). The descriptive mean differences ranged from 1.34 to 1.42 points. In both sampled scene sets, cognitive and emotional scores were numerically highest, whereas behavioral scores were lowest.

**Figure 5:**
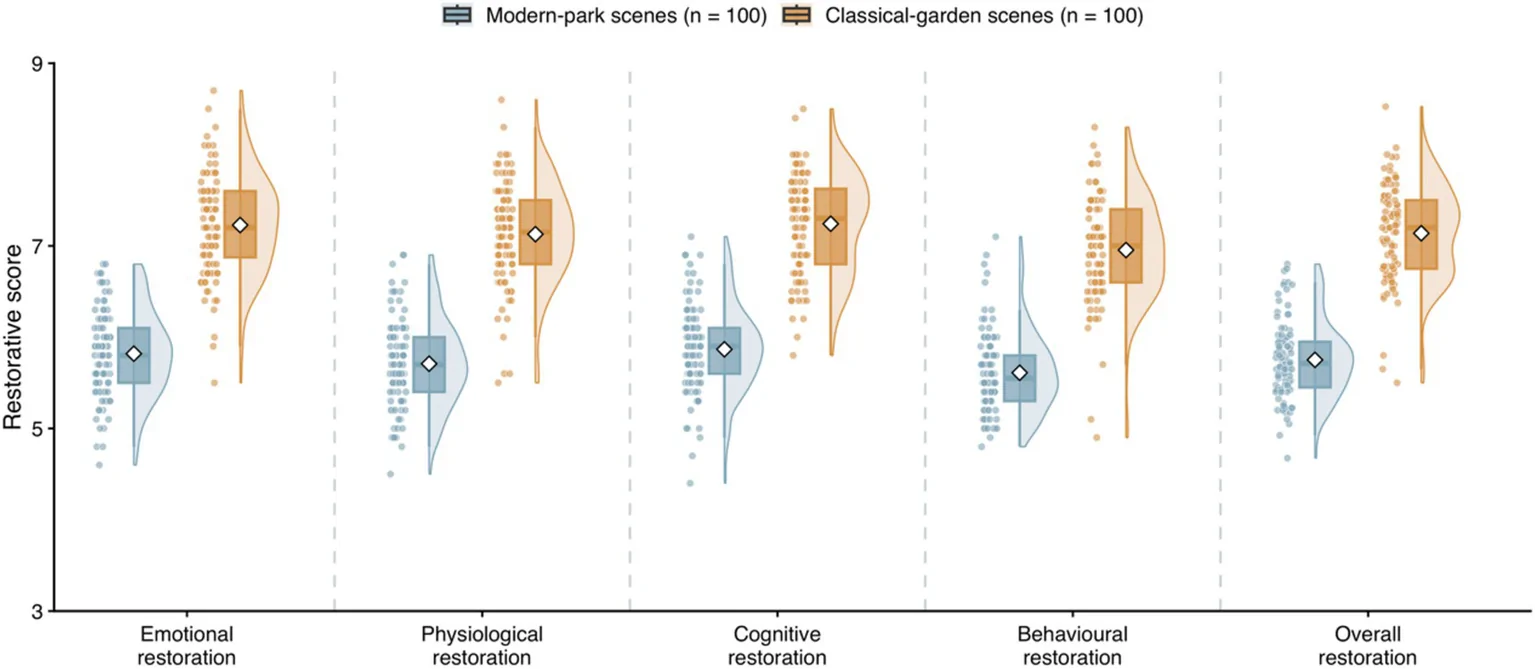
Perceived-restoration scores across the sampled scene sets. Points represent participant-average scores; half-violins show distributions. Boxes indicate medians and interquartile ranges; whiskers extend to observations within 1.5 interquartile ranges. White diamonds indicate means. *n* = participants.

Overall scores followed the same pattern, averaging 7.14 ± 0.52 for the sampled classical-garden scenes and 5.75 ± 0.42 for the sampled modern-park scenes, a descriptive difference of 1.39 points. Figure 5 shows higher central scores for the classical-garden scene set, although the participant-level distributions overlap. This pattern describes the particular scenes sampled in Suzhou and Xuzhou and cannot establish a general advantage of classical gardens over modern parks independently of city. Whether the lighting contrast differed between these scene sets was examined using the trial-level mixed-effects models reported in Section 3.2.

### 3.2 Differences in lighting responses between the sampled scene sets

Overall perceived-restoration scores showed different lighting-response patterns between the sampled scene sets (Figure 6a). The mixed-effects model estimated a landscape type × lighting interaction of −0.544 points (95% CI [−0.792, −0.295]; *t* (196) = −4.316, *p* < 0.001; Figure 6b and Supplementary Table S3). This interaction compares the artificial-lighting minus natural-light contrast in the sampled classical-garden scenes with the corresponding contrast in the sampled modern-park scenes.

**Figure 6:**
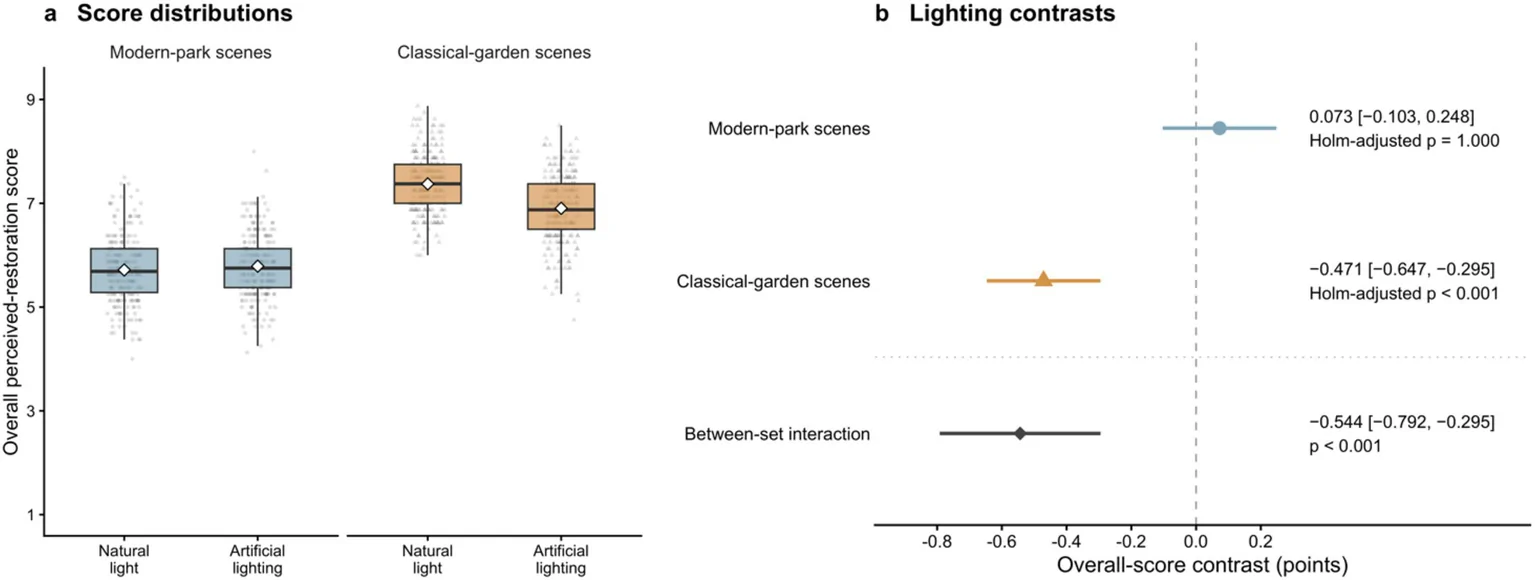
Overall perceived restoration under natural light and artificial lighting. **(a)** Points represent individual image evaluations; boxes show medians and interquartile ranges, whiskers extend to observations within 1.5 interquartile ranges, and white diamonds indicate means. **(b)** Points and horizontal lines show mixed-model contrasts and pointwise 95% confidence intervals, not SD or SE; the dashed line indicates zero. Analyses included 1,000 evaluations from 200 participants: 50 participants and 250 evaluations per condition, covering 10 base scenes, each paired across lighting conditions. The overall interaction was the single unadjusted primary test; within-set contrasts were Holm-adjusted within the family of 14 secondary tests.

For the sampled classical-garden scenes, estimated overall scores were 7.37 under natural light and 6.90 under artificial lighting, giving a lighting contrast of −0.471 points (95% CI [−0.647, −0.295]; Holm-adjusted *p* < 0.001). For the sampled modern-park scenes, the corresponding estimates were 5.72 and 5.79, with a contrast of 0.073 points (95% CI [−0.103, 0.248]; Holm-adjusted *p* = 1.000; Supplementary Table S5). Thus, artificial lighting was associated with lower overall scores in the sampled classical-garden scenes, whereas the estimate for the sampled modern-park scenes remained compatible with either a small decrease or an increase. The interaction supports a difference in lighting responses between these sampled scene sets, but cannot separate landscape type from city.

Model diagnostics included checks of convergence, singularity, residual-versus-fitted plots, *Q*–*Q* plots, and residual variability across conditions. Observations with absolute standardized residuals exceeding three were flagged for inspection but retained. Sensitivity of the primary interaction was examined by omitting each base scene in turn and by allowing scene-specific random slopes for lighting. Missing item responses were checked before analysis; none were identified, and no imputation was required.

### 3.3 Lighting-response patterns across the four restoration dimensions

As shown in Figure 7 and Supplementary Table S6, four dimension scores showed a consistent direction of interaction between the sampled scene sets. Interaction estimates were −0.498 points for emotional restoration (95% CI [−0.776, −0.220]; Holm-adjusted *p* = 0.0031), −0.642 for physiological restoration (95% CI [−0.914, −0.370]; adjusted *p* < 0.001), −0.506 for cognitive restoration (95% CI [−0.780, −0.232]; adjusted *p* = 0.0024), and −0.528 for behavioral restoration (95% CI [−0.797, −0.259]; adjusted *p* = 0.0012). All four interactions remained supported after correction across the 14 secondary tests. Although the physiological dimension had the largest numerical interaction estimate, this does not establish that its lighting response differed from those of the other dimensions.

**Figure 7:**
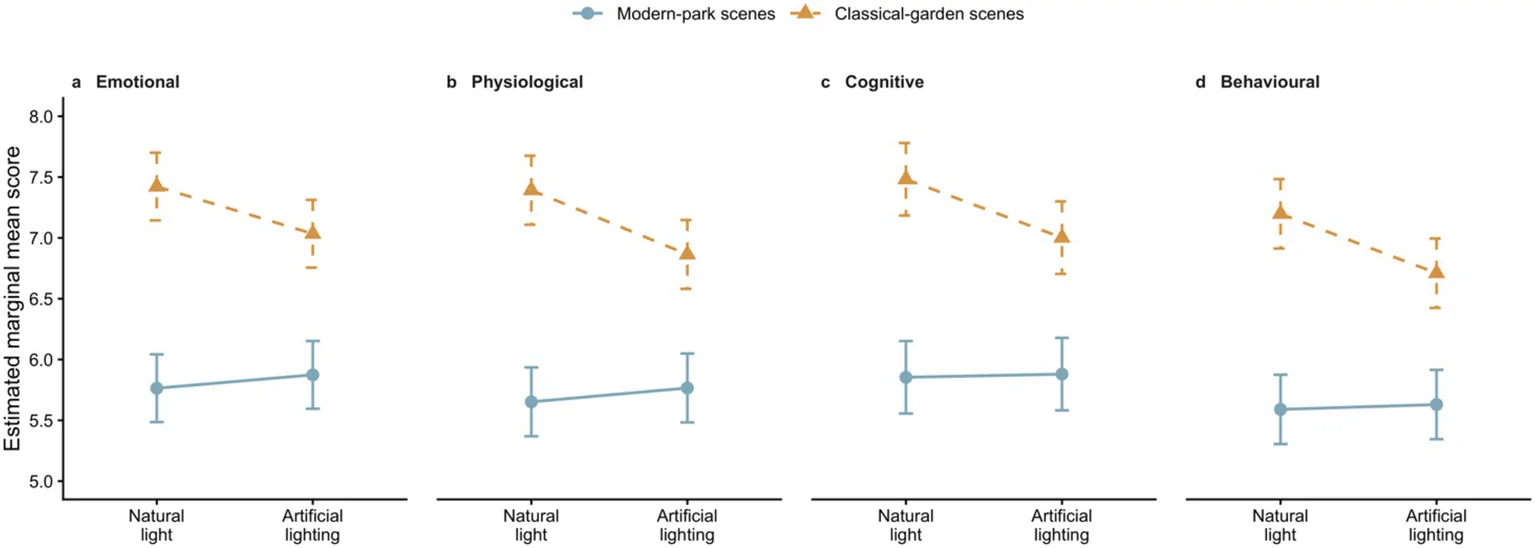
Dimension-specific perceived-restoration scores across lighting conditions. Error bars indicate 95% confidence intervals (CIs) for the estimated marginal means.

Within the sampled classical-garden scenes, artificial lighting was associated with lower scores across all four dimensions. Artificial-lighting minus natural-light contrasts were −0.388 points for emotional restoration, −0.528 for physiological restoration, −0.480 for cognitive restoration, and −0.488 for behavioral restoration (Supplementary Table S5). The emotional contrast had a Holm-adjusted *p* value of 0.0012, whereas the other three contrasts had adjusted *p* values below 0.001. Within the sampled modern-park scenes, corresponding contrasts were small and positive, ranging from 0.026 to 0.114 points; all confidence intervals included zero and all adjusted *p* values were 1.000.

These secondary results were directionally consistent with the overall-score interaction reported in Section 3.2. However, the dimension scores and overall score are mathematically related and do not constitute independent replications. Given the limited within-participant reliability and the unconfirmed four-dimensional structure, these findings should be interpreted as patterns in the original SRRS item groupings rather than evidence for distinct emotional, physiological, cognitive, or behavioral restoration mechanisms.

### 3.4 Associations between landscape characteristics and perceived-restoration outcomes

The image-level correlations showed several descriptive patterns (Supplementary Table S9). Vegetation coverage structure was positively correlated with overall perceived restoration across all four conditions (*r* = 0.566–0.938), as was vegetation maintenance (*r* = 0.410–0.798). Water-feature coefficients were positive in the sampled classical-garden scenes (*r* = 0.524–0.633) and close to zero in the sampled modern-park scenes (*r* = −0.104–0.017). Expert-rating agreement and uncertainty around image-level feature means are reported in Supplementary Tables S7, S8, respectively.

None of the 24 associations met the 0.05 threshold after Benjamini–Hochberg correction (minimum adjusted *p* = 0.230). Each correlation involved only five images, making its direction and magnitude potentially sensitive to individual scenes. These patterns therefore provide candidate hypotheses for studies with larger scene samples, but support no inferential conclusions about landscape predictors or differences between conditions.

## 4 Discussion

This study examined how landscape type, nighttime lighting, and observable landscape characteristics were associated with perceived restoration in nighttime green spaces. The central finding was that the lighting contrast differed between the sampled classical-garden and modern-park scene sets. Natural nighttime lighting was associated with higher self-reported scores in the sampled classical-garden scenes, whereas the estimate for the sampled modern-park scenes remained compatible with either a small decrease or an increase. The latter finding does not demonstrate equivalence between lighting conditions. Across lighting conditions, the classical-garden scene set also received higher descriptive ratings, but this comparison cannot separate landscape type from city or other characteristics of the sampled sites. The four dimension-specific patterns were consistent with the overall-score result, although they are mathematically related and do not constitute independent replications. The discussion therefore focuses on the interpretation of these scene-specific responses, possible perceptual explanations, and implications requiring further validation.

### 4.1 Differences in perceived restoration between the sampled scene sets

The consistently higher self-reported ratings for the sampled classical-garden scenes provide a starting point for examining how viewers evaluate different nighttime settings. They do not establish that classical gardens are inherently more restorative than modern parks or that landscape type is the strongest determinant of the responses. Prior studies have considered architectural variation, water, and cultural landscapes in relation to restorative evaluations, indicating that relevant qualities are not confined to a simple natural-versus-built classification (Lindal and Hartig, 2013; White et al., 2010; Xu et al., 2018). These findings help contextualize the present descriptive difference without identifying which characteristics explain it.

A plausible explanation lies in the distinctive spatial organization of classical garden landscapes. Unlike many contemporary urban parks, classical gardens are intentionally designed to create sequential visual experiences through the coordinated arrangement of vegetation, terrain, water, architectural structures, and enclosed spaces. This architectural description concerns a design tradition, not a manipulation or an explanation demonstrated by the sampled comparison. Rather than presenting the entire environment at once, these landscapes gradually reveal views through changes in perspective, framed scenery, and transitions between enclosed and relatively open spaces. Such spatial characteristics may provide sufficient visual richness to maintain involuntary attention while preserving overall coherence and legibility. Research on visual preference and perceived restorative potential provides a basis for considering complexity and coherence, although their relationships are not uniform across studies (Pazhouhanfar and Kamal, 2014; Stamps, 2004). From the perspective of Attention Restoration Theory, these qualities are likely to promote soft fascination, a sense of being away, and compatibility between the environment and the observer. At the same time, the harmonious integration of natural and built components may facilitate positive emotional responses and stress reduction, consistent with the mechanisms proposed by Stress Reduction Theory. These theoretical accounts frame hypotheses rather than demonstrated recovery processes in this study.

The arrangement of landscape features is consequently a useful topic for further investigation rather than an explanation established by the present findings. In classical gardens, built structures such as pavilions, corridors, bridges, and walls rarely function as isolated artificial elements. Instead, they are deliberately coordinated with vegetation, terrain, and water to guide movement, frame views, and organize spatial sequences. Consequently, artificial and natural elements may jointly contribute to spatial depth, visual continuity, and environmental legibility. This interpretation extends beyond an additive view of landscape perception, in which individual elements independently influence restoration, but remains a theoretical proposition here. This perspective is also consistent with previous studies considering perceived restoration in relation to landscape complexity, coherence, legibility, and the overall visual organization of a setting (Lindal and Hartig, 2013; Stamps, 2004; Xu et al., 2018). Visual-character frameworks likewise distinguish complexity, coherence, stewardship, and historicity as dimensions for describing landscapes rather than treating them as interchangeable measures of restorative response (Ode et al., 2008; Tveit et al., 2006).

The modern park scenes included in the present study generally exhibited more open layouts and less spatial layering. This observation motivates questions about how enclosure, visibility, and spatial sequence affect nighttime evaluations; it does not show that these scenes lack restorative qualities or that their openness caused lower ratings. The contribution of a feature cannot be identified from a comparison between two scene sets that also differ in city, site selection, and cultural context. Accordingly, the physical description of an image should remain separate from the psychological processes attributed to its viewer. Di Plinio et al. distinguish quantified environmental composition, perceived restorativeness, and affective states, providing a useful conceptual basis for this separation (Di Plinio et al., 2026a). Our expert scores describe observer-coded visual characteristics, while SRRS scores describe participants’ self-reported responses.

The intended interpretation of the SRRS also bounds the discussion. The original dimension labels are retained for consistency with Han (2003), but emotional scores describe reported feelings, physiological scores describe sensations associated with arousal, cognitive scores describe interest and perceived attention, and behavioral scores describe intentions to visit or stay. Lower endorsement of faster breathing and sweating is not a recording of physiological recovery. Similarly, interest is not demonstrated recovery of attentional capacity, and stated intentions are not subsequent behavior. The overall composite summarizes related perceived responses; it should not be interpreted as evidence of sustained mental-health improvement. This preserves the value of subjective evaluation without collapsing it into the broader restoration processes that would require baseline, change, or objective outcome measures.

### 4.2 Scene-specific effects of nighttime lighting

The contrasting lighting responses in the sampled scene sets suggest that perceived restoration should be considered in relation to scene content as well as illumination. Previous work has examined how the focus of light and the objects illuminated relate to perceived restorativeness, preference, and fear (Nikunen and Korpela, 2012, 2009). Other research considers perceived safety, pedestrian performance, and evaluations of outdoor lighting (Boomsma and Steg, 2014; Peña-García et al., 2015; Rahm and Johansson, 2018). These outcomes should not be treated as substitutes for restoration, but they demonstrate the range of perceptual and functional responses that lighting can elicit. A field experiment also found that the effect of additional ambient lighting on park preference varied with spatial context (Lis et al., 2024). Together, this literature supports evaluating specific lighting–scene combinations rather than assuming a universal benefit of additional light.

Classical garden landscapes can be described through layered spatial sequences, framed and partially concealed views, varied enclosure, and the integration of vegetation, terrain, water, and architecture. Under natural nighttime lighting, areas of light and shadow may preserve some of these relationships rather than revealing the entire scene simultaneously. Areas of light and shadow can maintain depth, ambiguity, and progressive visual disclosure. These perceptual qualities are compatible with the soft fascination and psychological disengagement discussed in restorative-environment theory. However, the present study did not quantify luminance transitions, visual adaptation, or perceived coherence. The explanation is therefore a candidate account of the sampled images, not evidence that natural lighting preserves the intended organization of classical gardens generally. Future work should measure these perceptual qualities alongside responses instead of inferring them from the lighting condition alone.

Artificial lighting may alter the perception of this spatial logic even when the physical composition of the landscape remains unchanged. Localized illumination may draw attention toward individual buildings, paths, trees, or decorative elements while reducing the perceptual continuity between them. These possibilities require direct testing; the present interaction does not demonstrate visual fragmentation or increased perceptual effort. Research on brightness perception and pedestrian responses emphasizes that evaluating lighting involves more than nominal illumination alone (Rahm and Johansson, 2018; Rea et al., 2011). The issue is therefore not whether artificial lighting is inherently beneficial or harmful, but whether its intensity, distribution, and visual emphasis are compatible with the landscape’s compositional structure. Here, the artificial-lighting stimuli were scene-specific simulations of existing site lighting, so neither the benefit nor the disadvantage can be assigned to a single lighting attribute.

The modern park scenes examined in this study generally presented more open, direct, and functionally legible spatial configurations. Paths, lawns, vegetation groups, and activity spaces can be visible as relatively independent components, with less reliance on concealment, sequential revelation, or subtle transitions between built and natural elements. Consequently, changes in nighttime lighting may modify the visibility of particular objects without necessarily changing the overall spatial reading of the landscape. This remains a possible explanation rather than evidence that the modern-park scenes were insensitive to illumination. The uncertain within-set contrast allows for more than one direction of response. Nor can the feature-level correlations establish that vegetation, maintenance, or terrain offset lighting effects. Such explanations would require a larger scene sample and explicit tests of the proposed pathways.

The contribution of nighttime lighting can thus be investigated relationally: through the fit between lighting appearance and landscape structure. A lighting condition that preserves the character of one scene may alter the experience of another. The concept of landscape–lighting compatibility offers a way to frame that question, but the present findings do not validate a Landscape Structure–Lighting Compatibility Framework. Research involving environmental walking and field assessment illustrates how responses can be evaluated while pedestrians encounter actual lighting environments (Patching et al., 2017; Rahm and Johansson, 2021). These methods could complement image-based experiments by testing whether perceived visual coherence, orientation, and willingness to remain explain responses to particular configurations. They would also help distinguish subjective scene evaluations from the physiological and attentional processes implied by a stronger restoration claim.

### 4.3 Implications for nighttime landscape design

The present findings motivate site-specific evaluation of nighttime landscape design rather than uniform assumptions about lighting benefits. As a design principle for testing, the interaction between lighting and spatial organization deserves attention. Accordingly, lighting design should begin with an understanding of the existing spatial organization of a site, including its hierarchy, depth, continuity, enclosure, and relationships among natural and built elements. Rather than treating lighting as an independent intervention, designers should coordinate the distribution, intensity, and visual emphasis of illumination with landscape structure so that lighting reinforces, rather than overrides, the intended spatial experience. These recommendations express objectives for assessment, not design effectiveness established by the present scene sample. Methods linking visual concepts to landscape attributes may help define what is being assessed before responses are compared (Ode et al., 2008; Tveit et al., 2006).

For the sampled classical-garden scenes, the lower ratings under the simulated artificial-lighting conditions motivate testing whether alternative configurations better preserve layered views, gradual spatial transitions, and the visual relationships among vegetation, water, terrain, and architecture. They do not justify a general recommendation to reduce or remove lighting from classical gardens. Likewise, the modern-park result does not establish that lighting is ineffective or that improving vegetation structure, maintenance, or terrain would increase perceived restoration. Across sites, lighting strategies may be evaluated according to how well they preserve spatial hierarchy, maintain visual continuity, minimize glare and excessive contrast, and support orientation without disrupting the coherence of the landscape. These criteria should be assessed rather than assumed to improve restoration. Studies of illuminance uniformity and brightness offer ways to characterize lighting appearance, but do not by themselves demonstrate restorative benefit (Narendran et al., 2016; Rea et al., 2011).

Such evaluation also requires separating subjective attractiveness from other design objectives. Evidence on woodland edges addresses safety and preference as distinct responses (Jorgensen et al., 2002), while park-lighting experiments show that spaces judged safest need not be those most conducive to contemplation or leisurely experience (Lis et al., 2024). Safety, accessibility, visual comfort, energy use, and ecological effects therefore remain necessary considerations when translating visual preferences into practice. The wider literature on artificial light at night raises human and ecosystem concerns that were not assessed in this experiment (Svechkina et al., 2020). A lower SRRS score for one simulated image cannot determine how these objectives should be balanced at an actual site.

The practical contribution is therefore to encourage context-sensitive comparisons of alternative designs, followed by field validation. Lighting can be considered as a tool for revealing landscape structure, but its effectiveness should be evaluated through actual visitor experience rather than inferred from image ratings alone. Photo-based screening can identify configurations for further investigation; it cannot specify optimal luminance, color temperature, or lighting schedules. The descriptive observations in the Supplementary material likewise cannot prescribe the addition or removal of individual landscape features. Their value is to guide better-sampled research questions, not to replace direct evaluation of a design intervention.

### 4.4 Limitations and future research

Several limitations should be considered when interpreting the findings. First, the study used static photographic stimuli, which could not reproduce movement, sound, social activity, or visual adaptation during an actual nighttime visit (Browning et al., 2020; Kjellgren and Buhrkall, 2010). The findings therefore concern self-reported responses rather than demonstrated recovery. Landscape type was also perfectly confounded with city, with only five base scenes per category. Although mixed-effects models accounted for participant and scene variability, they could not resolve this confounding or establish representativeness. Future studies should sample both landscape categories across multiple cities and combine image-based assessments with field evaluations.

Second, the artificial-lighting images were scene-specific simulations rather than photometrically calibrated or uniform manipulations. Consistent photographic procedures reduced some variation but did not ensure equivalent ambient illumination. The results consequently cannot isolate the effects of brightness, color temperature, glare, or lighting distribution. Future research should combine controlled lighting parameters with objective measurements and pedestrian assessments (Rahm and Johansson, 2021), while treating landscape–lighting compatibility as a hypothesis requiring direct testing.

Finally, the adapted SRRS showed limited within-participant reliability, and its four-dimensional structure was not confirmed; the scores should therefore be interpreted cautiously as self-reported evaluations. Cultural and identity-related processes may also shape environmental responses (Di Plinio et al., 2026b; Morton et al., 2017; Ren, 2019; Yang and Brown, 1992). Familiarity, cultural identification, connectedness to nature, current stress or fatigue, and previous nighttime-park experience were not measured. These factors warrant assessment in future studies. The analytical hierarchy was established during revision, and the supplementary feature correlations remain descriptive rather than evidence of landscape predictors.

## 5 Conclusion

This study investigated how nighttime lighting was associated with perceived restoration across five classical-garden scenes from Suzhou and five modern-park scenes from Xuzhou. Trial-level mixed-effects analyses accounting for participant and base-scene variability identified a landscape set × lighting interaction in overall perceived restoration. Artificial lighting was associated with lower scores than natural nighttime lighting in the sampled classical-garden scenes, whereas no statistically detectable difference was found in the sampled modern-park scenes. The latter finding does not establish equivalence between lighting conditions. These results are restricted to the sampled scenes. Landscape set was completely confounded with city, and the scene-specific lighting simulations do not support general conclusions about classical gardens, modern parks, or artificial lighting. Outcomes represent self-reported responses to images rather than demonstrated recovery in real environments. Dimension-specific findings were secondary, and feature-level correlations remained descriptive and supported no inferential conclusions.

The findings motivate further investigation of how lighting and scene composition jointly relate to perceived restoration. Studies sampling both landscape categories across multiple cities, using larger scene sets and validated lighting manipulations, are needed before deriving design recommendations. Stronger psychometric evaluation and field-based measures of restoration would further clarify the practical relevance of these observations.

## Data Availability

The original contributions presented in the study are included in the article/Supplementary material, further inquiries can be directed to the corresponding authors.
