# Supplementary material for: Nighttime lighting effects on perceived restoration depend on landscape type: evidence from classical garden landscapes and modern park landscapes
Source: Front Psychol. 2026 Oct 23;17:1958901. doi: 10.3389/fpsyg.2026.1958901 (PMC13760590; doi:10.3389/fpsyg.2026.1958901)
Supplement: Supplementary file 1 [file Table_1.DOCX]

**Table S1. Expert Rating Criteria for Landscape Characteristics**

| Landscape characteristic | 1 | 2 | 3 |
| --- | --- | --- | --- |
| Proportion of artificial elements | Artificial elements occupy 0–20% of the image | Artificial elements occupy 20–50% of the image | Artificial elements occupy more than 50% of the image |
| Water features | No visible water features | Water features occupy a relatively small part of the image | Water features occupy a relatively large part of the image |
| Vegetation coverage structure | A single vegetation layer or type | A two-layer vegetation structure, such as trees and shrubs or trees and ground cover | A multilayered vegetation structure comprising trees, shrubs, and ground cover |
| Spatial openness | Predominantly enclosed space with limited visual depth | Semi-enclosed or semi-open space | Predominantly open space with extensive visual depth |
| Vegetation maintenance | Low level of maintenance, with relatively unmanaged vegetation | Moderate level of maintenance | High level of maintenance, with vegetation appearing well managed |
| Terrain undulation | Predominantly flat terrain | Slightly undulating terrain | Strongly undulating terrain with evident elevation changes |

Note: Experts were instructed to rate only the landscape characteristics visible in each image and not to evaluate its perceived restoration. Each of the 20 images was assessed independently using the three-point ordinal scale shown above. Higher scores indicate a greater proportion, complexity, openness, maintenance level, or degree of terrain undulation, depending on the characteristic.

**Table S2. Original English SRRS items and Chinese items administered in this study.**

| Dimension in Han (2003) | Item | Original English wording | Chinese wording administered | Scoring |
| --- | --- | --- | --- | --- |
| Emotional | Q1 | Good natured | 我的心情很愉悦 | Direct |
|  | Q2 | Relaxed | 我的心情很放松 | Direct |
| Physiological | Q3 | My breathing is becoming faster | 我的呼吸变得急促 | Reverse |
|  | Q4 | My hands are sweating | 我的手心在出汗 | Reverse |
| Cognitive | Q5 | I am interested in the presented scene | 我对这个场景很感兴趣 | Direct |
|  | Q6 | I feel attentive to the presented scene | 我十分专注地欣赏该场景 | Direct |
| Behaviour | Q7 | I would like to visit here more often | 我想经常到这里游览 | Direct |
|  | Q8 | I would like to stay here longer | 我想在这里多呆一会 | Direct |

Note: Q1–Q8 correspond to items 1–8 in the administered Chinese questionnaire. Responses were scored from 1 to 9; Q3 and Q4 were reverse-scored as 10 minus the original response.

**Table S3a. Within-participant inter-item correlations and internal consistency of the adapted SRRS scores.**

| Score | Inter-item correlation, *r* [95% CI] | Spearman–Brown coefficient  [95% CI] | Cronbach’s α  [95% CI] | McDonald’s ω  [95% CI] |
| --- | --- | --- | --- | --- |
| Emotional | 0.242 [0.183, 0.297] | 0.390 [0.309, 0.458] | — | — |
| Physiological | 0.253 [0.186, 0.319] | 0.404 [0.314, 0.484] | — | — |
| Cognitive | 0.189 [0.125, 0.255] | 0.318 [0.222, 0.406] | — | — |
| Behavioural | 0.156 [0.090, 0.223] | 0.270 [0.166, 0.365] | — | — |
| Overall composite | — | — | 0.662 [0.621, 0.697] | 0.669 [0.631, 0.707] |

**Table S3b. Dimensional-structure assessment and sensitivity analysis of the overall composite.**

| Model | Scaled χ² (df) | p | Robust CFI | Robust RMSEA | SRMR within | Assessment |
| --- | --- | --- | --- | --- | --- | --- |
| One within-participant factor; unrestricted between-participant covariance | 15.480 (20) | .748 | 1.000 | .000 | .017 | Admissible solution |
| Four correlated within-participant factors; unrestricted between-participant covariance | 12.902 (14) | .534 | 1.000 | .000 | .015 | Inadmissible latent covariance matrix |
| One within-participant factor after adjustment for image-specific item means; unrestricted between-participant covariance | 17.949 (20) | .591 | 1.000 | .000 | .020 | Admissible sensitivity model |

Note: Brackets indicate 95% confidence intervals; dashes indicate statistics not reported. Fit indices for the inadmissible four-factor model are provided for transparency and should not be interpreted as evidence supporting that structure.

**Table S4. Descriptive summary of perceived-restoration scores across the sampled scene sets.**

| **Outcome** | **Sampled classical-garden scenes (n = 100)** | **Sampled modern-park scenes (n = 100)** | **Descriptive mean difference** |
| --- | --- | --- | --- |
| Emotional | 7.23 ± 0.56 | 5.82 ± 0.47 | 1.41 |
| Physiological | 7.13 ± 0.56 | 5.71 ± 0.48 | 1.42 |
| Cognitive | 7.24 ± 0.55 | 5.87 ± 0.49 | 1.38 |
| Behavioural | 6.95 ± 0.57 | 5.61 ± 0.44 | 1.34 |
| Overall | 7.14 ± 0.52 | 5.75 ± 0.42 | 1.39 |

Note: Values are means ± SD of participants’ five-image average scores, pooled across natural light and artificial lighting. Each scene set included 100 participants. Descriptive mean differences were calculated from unrounded values as sampled classical-garden scenes minus sampled modern-park scenes.

**Table S5. Model-estimated lighting contrasts for perceived-restoration outcomes within the sampled scene sets.**

| **Sampled scene set** | **Outcome** | **Artificial lighting, EMM** | **Natural light, EMM** | **Difference [95% CI]** | **t (df)** | **Holm-adjusted p** |
| --- | --- | --- | --- | --- | --- | --- |
| Classical-garden | Emotional | 7.034 | 7.422 | −0.388 [−0.585, −0.191] | −3.890 (196) | 0.0012 |
| Classical-garden | Physiological | 6.864 | 7.392 | −0.528 [−0.720, −0.336] | −5.415 (196) | <0.001 |
| Classical-garden | Cognitive | 7.002 | 7.482 | −0.480 [−0.673, −0.287] | −4.894 (196) | <0.001 |
| Classical-garden | Behavioural | 6.710 | 7.198 | −0.488 [−0.679, −0.297] | −5.051 (196) | <0.001 |
| Classical-garden | Overall | 6.903 | 7.374 | −0.471 [−0.647, −0.295] | −5.290 (196) | <0.001 |
| Modern-park | Emotional | 5.874 | 5.764 | 0.110 [−0.087, 0.307] | 1.103 (196) | 1.000 |
| Modern-park | Physiological | 5.766 | 5.652 | 0.114 [−0.078, 0.306] | 1.169 (196) | 1.000 |
| Modern-park | Cognitive | 5.880 | 5.854 | 0.026 [−0.167, 0.219] | 0.265 (196) | 1.000 |
| Modern-park | Behavioural | 5.630 | 5.590 | 0.040 [−0.151, 0.231] | 0.414 (196) | 1.000 |
| Modern-park | Overall | 5.788 | 5.715 | 0.073 [−0.103, 0.248] | 0.814 (196) | 1.000 |

Note: EMM = estimated marginal mean; CI = confidence interval. Estimates were obtained from trial-level mixed-effects models with landscape type, lighting, and their interaction as fixed effects and participant and base-scene random intercepts. Differences are artificial lighting minus natural light, with Kenward–Roger degrees of freedom and pointwise 95% CIs. Holm adjustment covered all 14 secondary tests: the ten lighting contrasts shown here and four dimension-specific interactions. Estimates were calculated before rounding.

**Table S6.** **Landscape-by-lighting interaction contrasts for the four SRRS dimensions.**

| **Outcome** | **Interaction estimate** | **SE** | **95% CI** | **t (df)** | **Holm-adjusted p** |
| --- | --- | --- | --- | --- | --- |
| Emotional | −0.498 | 0.141 | [−0.776, −0.220] | −3.530 (196) | 0.0031 |
| Physiological | −0.642 | 0.138 | [−0.914, −0.370] | −4.656 (196) | <0.001 |
| Cognitive | −0.506 | 0.139 | [−0.780, −0.232] | −3.648 (196) | 0.0024 |
| Behavioural | −0.528 | 0.137 | [−0.797, −0.259] | −3.864 (196) | 0.0012 |

Note: SE = standard error; CI = confidence interval; df = degrees of freedom. Holm-adjusted p values account for 14 secondary tests.

**Table S7. Agreement among experts on ordinal landscape-characteristic ratings.**

| Landscape characteristic | Mean pairwise weighted *κ* | 95% CI |
| --- | --- | --- |
| Artificial elements | 0.460 | [0.262, 0.594] |
| Water features | 0.852 | [0.674, 0.889] |
| Vegetation coverage structure | 0.335 | [0.028, 0.431] |
| Spatial openness | 0.522 | [0.269, 0.641] |
| Vegetation maintenance | 0.489 | [0.401, 0.548] |
| Terrain undulation | 0.700 | [0.417, 0.812] |

Note: κ is the mean of 105 pairwise linearly weighted Cohen’s kappa coefficients among 15 experts rating 20 images. CI = confidence interval; approximate 95% CIs were obtained from 1,000 bootstrap resamples of the ten paired base scenes.

**Table S8. Image-level mean expert ratings and their 95% confidence intervals.**

| **Image ID** | **Scene set** | **Lighting** | **Artificial elements** | **Water features** | **Vegetation structure** | **Openness** | **Maintenance** | **Terrain** |
| --- | --- | --- | --- | --- | --- | --- | --- | --- |
| **1** | Modern-park | Natural light | 1.87 [1.67, 2.00] | 1.00 [1.00, 1.00] | 2.20 [1.93, 2.47] | 2.87 [2.67, 3.00] | 1.80 [1.47, 2.13] | 1.07 [1.00, 1.20] |
| 2 | Modern-park | Artificial lighting | 1.93 [1.67, 2.27] | 1.07 [1.00, 1.20] | 1.87 [1.67, 2.00] | 2.87 [2.67, 3.00] | 3.00 [3.00, 3.00] | 1.07 [1.00, 1.20] |
| 3 | Modern-park | Natural light | 1.20 [1.00, 1.40] | 1.00 [1.00, 1.00] | 2.87 [2.67, 3.00] | 1.80 [1.60, 2.00] | 1.80 [1.60, 2.00] | 1.00 [1.00, 1.00] |
| 4 | Modern-park | Artificial lighting | 2.00 [1.73, 2.27] | 1.07 [1.00, 1.20] | 2.67 [2.40, 2.87] | 2.13 [1.87, 2.40] | 2.87 [2.67, 3.00] | 1.00 [1.00, 1.00] |
| 5 | Modern-park | Natural light | 2.07 [1.80, 2.33] | 1.00 [1.00, 1.00] | 2.93 [2.80, 3.00] | 2.00 [2.00, 2.00] | 2.80 [2.60, 3.00] | 2.87 [2.67, 3.00] |
| 6 | Modern-park | Artificial lighting | 2.20 [1.93, 2.47] | 1.07 [1.00, 1.20] | 2.73 [2.47, 2.93] | 1.73 [1.47, 1.93] | 2.93 [2.80, 3.00] | 2.87 [2.67, 3.00] |
| 7 | Modern-park | Natural light | 1.13 [1.00, 1.33] | 1.00 [1.00, 1.00] | 1.93 [1.60, 2.27] | 2.73 [2.47, 2.93] | 2.27 [2.07, 2.47] | 1.07 [1.00, 1.20] |
| 8 | Modern-park | Artificial lighting | 2.13 [2.00, 2.33] | 1.07 [1.00, 1.20] | 1.87 [1.53, 2.20] | 2.67 [2.40, 2.87] | 1.93 [1.67, 2.20] | 1.07 [1.00, 1.20] |
| 9 | Modern-park | Natural light | 1.07 [1.00, 1.20] | 2.13 [2.00, 2.33] | 2.13 [1.87, 2.40] | 1.07 [1.00, 1.20] | 2.13 [2.00, 2.33] | 1.07 [1.00, 1.20] |
| 10 | Modern-park | Artificial lighting | 2.00 [1.80, 2.20] | 2.87 [2.67, 3.00] | 2.60 [2.33, 2.87] | 1.20 [1.00, 1.40] | 2.00 [1.73, 2.27] | 1.00 [1.00, 1.00] |
| 11 | Classical-garden | Natural light | 1.87 [1.60, 2.13] | 1.00 [1.00, 1.00] | 2.80 [2.60, 3.00] | 1.93 [1.67, 2.20] | 1.80 [1.53, 2.07] | 1.07 [1.00, 1.20] |
| 12 | Classical-garden | Artificial lighting | 1.93 [1.73, 2.13] | 1.00 [1.00, 1.00] | 2.87 [2.67, 3.00] | 2.00 [1.67, 2.33] | 2.87 [2.67, 3.00] | 1.00 [1.00, 1.00] |
| 13 | Classical-garden | Natural light | 1.93 [1.73, 2.13] | 2.80 [2.60, 3.00] | 2.80 [2.60, 3.00] | 2.07 [2.00, 2.20] | 2.87 [2.67, 3.00] | 1.13 [1.00, 1.33] |
| 14 | Classical-garden | Artificial lighting | 2.67 [2.40, 2.87] | 2.87 [2.67, 3.00] | 2.73 [2.53, 2.93] | 2.07 [1.87, 2.27] | 3.00 [3.00, 3.00] | 1.00 [1.00, 1.00] |
| 15 | Classical-garden | Natural light | 2.13 [1.87, 2.40] | 1.20 [1.00, 1.40] | 2.80 [2.60, 3.00] | 1.20 [1.00, 1.40] | 1.93 [1.73, 2.13] | 2.27 [1.93, 2.53] |
| 16 | Classical-garden | Artificial lighting | 1.93 [1.73, 2.13] | 1.00 [1.00, 1.00] | 2.80 [2.60, 3.00] | 1.07 [1.00, 1.20] | 2.93 [2.80, 3.00] | 2.00 [1.80, 2.20] |
| 17 | Classical-garden | Natural light | 2.87 [2.67, 3.00] | 1.00 [1.00, 1.00] | 2.80 [2.60, 3.00] | 1.20 [1.00, 1.40] | 2.13 [1.80, 2.47] | 1.07 [1.00, 1.20] |
| 18 | Classical-garden | Artificial lighting | 2.73 [2.47, 2.93] | 1.00 [1.00, 1.00] | 3.00 [3.00, 3.00] | 1.47 [1.20, 1.73] | 2.93 [2.80, 3.00] | 1.07 [1.00, 1.20] |
| 19 | Classical-garden | Natural light | 2.20 [1.93, 2.47] | 2.80 [2.60, 3.00] | 2.93 [2.80, 3.00] | 1.93 [1.67, 2.13] | 2.87 [2.67, 3.00] | 2.13 [1.87, 2.40] |
| 20 | Classical-garden | Artificial lighting | 3.00 [3.00, 3.00] | 3.00 [3.00, 3.00] | 2.93 [2.80, 3.00] | 2.13 [1.87, 2.40] | 2.93 [2.80, 3.00] | 1.67 [1.40, 2.00] |

Note: Values are mean [95% CI] from 15 expert ratings per image on a three-category scale (1–3). CI = confidence interval; pointwise percentile CIs were obtained from 2,000 expert-level bootstrap resamples. Vegetation structure, openness, maintenance, and terrain denote vegetation coverage structure, spatial openness, vegetation maintenance, and terrain undulation, respectively.

**Table S9. Descriptive image-level correlations between landscape characteristics and overall perceived restoration.**

| **Sampled scene set** | **Lighting** | **Landscape characteristic** | **n** | **Pearson’s r** | **Unadjusted p** | **BH-adjusted p** | **Sampled scene set** |
| --- | --- | --- | --- | --- | --- | --- | --- |
| Classical-garden | Natural light | Artificial elements | 5 | 0.469 | 0.426 | 0.708 | Classical-garden |
| Classical-garden | Natural light | Water features | 5 | 0.633 | 0.252 | 0.701 | Classical-garden |
| Classical-garden | Natural light | Vegetation coverage structure | 5 | 0.578 | 0.308 | 0.701 | Classical-garden |
| Classical-garden | Natural light | Spatial openness | 5 | 0.228 | 0.712 | 0.917 | Classical-garden |
| Classical-garden | Natural light | Vegetation maintenance | 5 | 0.798 | 0.106 | 0.532 | Classical-garden |
| Classical-garden | Natural light | Terrain undulation | 5 | −0.092 | 0.883 | 0.922 | Classical-garden |
| Classical-garden | Artificial lighting | Artificial elements | 5 | 0.936 | 0.019 | 0.230 | Classical-garden |
| Classical-garden | Artificial lighting | Water features | 5 | 0.524 | 0.365 | 0.701 | Classical-garden |
| Classical-garden | Artificial lighting | Vegetation coverage structure | 5 | 0.566 | 0.320 | 0.701 | Classical-garden |
| Classical-garden | Artificial lighting | Spatial openness | 5 | 0.186 | 0.765 | 0.917 | Classical-garden |
| Classical-garden | Artificial lighting | Vegetation maintenance | 5 | 0.410 | 0.493 | 0.739 | Classical-garden |
| Classical-garden | Artificial lighting | Terrain undulation | 5 | 0.099 | 0.874 | 0.922 | Classical-garden |
| Modern-park | Natural light | Artificial elements | 5 | 0.288 | 0.638 | 0.900 | Modern-park |
| Modern-park | Natural light | Water features | 5 | −0.104 | 0.868 | 0.922 | Modern-park |
| Modern-park | Natural light | Vegetation coverage structure | 5 | 0.836 | 0.078 | 0.532 | Modern-park |
| Modern-park | Natural light | Spatial openness | 5 | −0.454 | 0.443 | 0.708 | Modern-park |
| Modern-park | Natural light | Vegetation maintenance | 5 | 0.684 | 0.203 | 0.701 | Modern-park |
| Modern-park | Natural light | Terrain undulation | 5 | 0.791 | 0.111 | 0.532 | Modern-park |
| Modern-park | Artificial lighting | Artificial elements | 5 | 0.197 | 0.750 | 0.917 | Modern-park |
| Modern-park | Artificial lighting | Water features | 5 | 0.017 | 0.979 | 0.979 | Modern-park |
| Modern-park | Artificial lighting | Vegetation coverage structure | 5 | 0.938 | 0.018 | 0.230 | Modern-park |
| Modern-park | Artificial lighting | Spatial openness | 5 | −0.602 | 0.283 | 0.701 | Modern-park |
| Modern-park | Artificial lighting | Vegetation maintenance | 5 | 0.510 | 0.380 | 0.701 | Modern-park |
| Modern-park | Artificial lighting | Terrain undulation | 5 | 0.527 | 0.361 | 0.701 | Modern-park |

Note: n = images; BH = Benjamini–Hochberg correction across all 24 associations. Image means were based on 15 expert ratings for landscape characteristics and 50 participant evaluations for overall scores. Results are descriptive and hypothesis-generating; no inferential conclusion is supported.
